# Effect of *HVEM/CD160* Variations on the Clear Cell Renal Carcinoma Risk and Overall Survival

**DOI:** 10.3390/ijms25136860

**Published:** 2024-06-22

**Authors:** Anna Andrzejczak, Bartosz Małkiewicz, Krzysztof Tupikowski, Kuba Ptaszkowski, Tomasz Szydełko, Lidia Karabon

**Affiliations:** 1 Laboratory of Genetic and Epigenetic of Human Diseases, Department of Experimental Therapy, Hirszfeld Institute of Immunology and Experimental Therapy, Polish Academy of Sciences, 53-114 Wroclaw, Poland; anna.andrzejczak@hirszfeld.pl; 2 Department of Minimally Invasive and Robotic Urology, University Center of Excellence in Urology, Wroclaw Medical University, 50-556 Wroclaw, Poland; bartosz.malkiewicz@umw.edu.pl (B.M.); tomasz.szydelko@umw.edu.pl (T.S.); 3 Subdivision of Urology, Lower Silesian Center for Oncology, Pulmonology and Hematology, 53-413 Wroclaw, Poland; krzysztof.tupikowski@dcopih.pl; 4 Department of Clinical Biomechanics and Physiotherapy in Motor System Disorders, Wroclaw Medical University, 50-368 Wroclaw, Poland; kuba.ptaszkowski@umw.edu.pl

**Keywords:** HVEM, CD160, clear cell renal cell carcinoma (ccRCC), single nucleotide gene polymorphism (SNP), immune checkpoint, disease risk, overall survival

## Abstract

Renal cell carcinoma (RCC) accounts for approximately 90–95% of all kidney cancers in adults, with clear cell RCC (ccRCC) being the most frequently identified subtype. RCC is known for its responsiveness to immunotherapy, making it an area of significant research interest. Immune checkpoint (IC) molecules, which regulate immune surveillance, are established therapeutic targets in RCC. The aim of this study was to analyze the influence of *HVEM* and *CD160* gene polymorphisms on ccRCC susceptibility and patient overall survival (OS) over a ten-year period of observation. We genotyped three *HVEM* single nucleotide polymorphisms (SNPs): rs1886730, rs2234167, and rs8725, as well as two *CD160* SNPs: rs744877 and rs2231375, in 238 ccRCC patients and 521 controls. Our findings indicated that heterozygosity within rs2231375 and/or rs2234167 increases ccRCC risk. Furthermore, in women, heterozygosity within *HVEM* SNPs rs8725 and rs1886730 is also associated with an increased ccRCC risk. The presence of a minor allele for rs1886730, rs2234167, rs8725, and rs2231375 was also correlated with certain clinical features of ccRCC. Moreover, rs1886730 was found to be associated with OS. In conclusion, our study highlights an association between *HVEM* and CD160 polymorphisms and the risk of developing ccRCC as well as OS.

## 1. Introduction

Renal cell carcinoma (RCC) is a common and deadly disease. In 2020, there were an estimated 431,288 new cases of RCC globally, of which 138,611 were in Europe, while worldwide mortality from RCC was 179,368 deaths (115,600 men and 63,768 women) [1]. Clear cell renal carcinoma (ccRCC) is the most common subtype of RCC in adults, accounting for around 80% of all RCC cases [2]. Named for its characteristic clear cytoplasm visible under microscopic examination, ccRCC typically originates from the epithelial cells of the proximal tubule in the renal cortex [3]. This subtype is known for its rapid and expansive growth, often resulting in an advanced stage at diagnosis. The elevated mortality rate associated with ccRCC can be attributed, in part, to its asymptomatic nature during the early stages of the disease, often leading to the development of metastatic tumors [2]. In 2022 alone, ccRCC contributed to 155,700 deaths, emphasizing its substantial impact on mortality rates in genitourinary cancers [4]. Given the absence of dependable early diagnostic markers for ccRCC, it is still necessary to discover new diagnostic and prognostic indicators for this condition.

Immune checkpoints (ICs) are pivotal regulators, maintaining immune equilibrium by fine-tuning the strength and duration of immune responses. Cancer cells exploit the checkpoints’ ability to downregulate the immune response by overexpressing them on the cell surface, enabling evasion of immune surveillance [5]. Harnessing this knowledge has led to the development of immunotherapies, like immune checkpoint inhibitors, which enhance the body’s ability to recognize and eliminate cancer cells, revolutionizing cancer treatment approaches [6]. Among them, the herpesvirus entry mediator (HVEM) and CD160 are important immune checkpoints, orchestrating complex regulatory roles in immune responses [7].

The HVEM, a member of the tumor necrosis factor (TNF) receptor superfamily, plays a dual role in immune modulation. The HVEM is present on the surface of various immune cells, including T cells, B cells, NK cells, and dendritic cells, as well as endothelial cells. The HVEM interacts with multiple ligands, including LIGHT, LTα (Lymphotoxin-alpha), BTLA (B- and T-lymphocyte attenuator), and CD160, and provides stimulatory or inhibitory signaling [8,9]. CD160 is a glycoprotein, a member of the immunoglobulin superfamily (IgSF), expressed on the surface of various immune cells, including T cells, NK cells, and some B cells. CD160 has a dual role, acting as a co-stimulatory molecule, binding to major histocompatibility complex class I (MHC I), and as a co-inhibitory molecule, binding to the HVEM and impacting the intensity of immune responses. The HVEM/CD160 interaction is a rare example of a direct interaction between the IgSF and TNF superfamily that contributes to the complex network of immune checkpoint regulation, negatively affecting the activation, proliferation, and cytokine production of different immune cell types [7]. The intricate interplay between the HVEM and CD160 underscores the complexity of immune regulation. Aberrant expression of the HVEM has been observed in cancer, occurring on either tumor-infiltrating lymphocytes (TILs) or tumor cells, thereby suppressing immune responses and promoting immune evasion [10,11,12]. Similarly, CD160 expression has been identified in specific cancer types, including hematological malignancies and solid tumors. Numerous studies propose its role in tumor progression, immune evasion, and potential implications for cancer therapy [13,14,15], highlighting the significance of the HVEM and CD160 in cancer biology.

Given the above-mentioned literature data, it can be hypothesized that ccRCC risk, as well as the clinical course of the disease, depends on the expression level of ICs, inter alia members of the HVEM/CD160 axis. It is well established that the mRNA and protein expression level can be regulated, among others, by genetic variations that can affect epigenetic modifications (methylation, microRNA binding), transcription factors’ binding sites, and the formation of protein isoforms [16]. Therefore, we put forward the hypothesis that single nucleotide polymorphisms (SNPs) located within genes encoding the HVEM and CD160 molecules, as well as SNP–SNP interactions (between variations in genes encoding the receptor and ligand), are associated with ccRCC risk and outcomes.

To date, only a limited number of studies have investigated the implications of *HVEM* polymorphisms in the context of cancer [17,18]. The potential roles of *HVEM* SNPs and their associations with malignancies remain unclear. Furthermore, there is a notable lack of literature regarding *CD160* gene variation and its potential relevance to cancer. Addressing the aforementioned gap in knowledge, our study specifically delved into examining the correlation between specific variants of *HVEM* and *CD160* genes and their potential influence on susceptibility to ccRCC as well as ccRCC patients’ overall survival (OS).

## 2. Results

### 2.1. Correlation of HVEM and CD160 Gene SNPs with ccRCC Susceptibility

The genotype distribution of all SNPs was within the Hardy–Weinberg equilibrium (HWE) in the control group. In the patient group, a deviation from the HWE was observed for *CD160* rs2231375 (f = −0.15; *p* = 0.02).

Table 1 presents the frequencies of genotypes of all studied SNPs in the *HVEM* and *CD160* genes for ccRCC patients and healthy controls (HCs). In the overall analysis, we found that rs2234167 (*HVEM*) and rs2231375 (*CD160*) might be associated with susceptibility to ccRCC. For the investigated *HVEM* SNPs, we noticed that the overall genotype distribution of rs2234167 differed between patients and HCs (*p* = 0.057). Moreover, by analyzing the particular genotypes, we noted that the frequency of the carriers of the A allele (AG and AA genotypes) was significantly higher in patients compared to in HCs and that those patients had around 1.5 times higher odds of ccRCC development as compared to the GG individuals (OR = 1.47; 95% CI 1.04–2.07; *p* = 0.03). We also noticed a higher frequency of the rs2234167 AG heterozygotes among patients (28.57% vs. 20.73%), and the individuals bearing that genotype had higher odds of developing ccRCC compared to the GG homozygous individuals (AG vs. GG, OR = 1.53; 95% CI 1.08–2.18; *p* = 0.02). Additionally, we observed an overrepresentation of ccRCC patients carrying the C allele (CT and CC genotypes) in rs1886730 (OR = 1.41; 95% CI 0.98–2.04; *p* = 0.06) as well as patients carrying the A allele (AG and AA genotypes) in rs8725 (OR = 1.37; 95% CI 0.96–1.97, *p* = 0.08).

For *CD160* SNPs, we observed that the genotype distribution of rs2231375 significantly differed between cases and HCs (*p* = 0.03). Interestingly, only CT heterozygotes were markedly overrepresented among patients (55.08% vs. 49.52%), but not recessive TT homozygotes. Hence, the CT genotype increased the risk of developing ccRCC compared to subjects with the CC and TT genotypes (CT vs. CC, OR= 1.47; 95% CI 1.05–2.06; *p* = 0.02, CT vs. TT, OR = 1.61; 95% CI 1.00–2.61; *p* = 0.05, CT vs. CC + TT, OR 1.51, 95% CI 1.08–2.06, *p* = 0.01). For the rs744877 SNP, we did not observe any association with susceptibility to ccRCC in the overall analysis.

The Svejgaard and Ryder method [19] was employed to assess the significance of the potential influence of the two genetic factors that were observed to be associated with ccRCC risk. The following factors were considered: factor A—heterozygosity of *HVEM* rs2243167 [AG] A+; factor B—heterozygosity of *CD160* rs2231375 [CT] B+. The results of that analysis are presented in Table 2. The frequency of carrying susceptible genotypes for both SNPs (factor A and factor B) was significantly higher in the ccRCC patients compared to in the individuals lacking factor A (A−) and B (B−) (test [h]). Possessing both factors A and B increased the odds of disease two-fold (OR 2.07, 95% CI 1.30–3.35, *p* = 0.003). In addition, we observed that the presence of one of the mentioned factors, A [d] or B [f], significantly increased the odds of ccRCC as compared to individuals not carrying any susceptible genotypes (OR 1.85, 95% CI 1.09–3.13, *p* = 0.02 and OR 1.56, 95% CI 1.09–2.23, *p* = 0.02, respectively).

### 2.2. Multifactorial Regression Analysis

Based on univariate analysis regarding the influence of the genetic variations of *HVEM* and *CD160* genes investigated on ccRCC susceptibility, a multivariate logistic regression model was established. Similar to the results of the univariate analysis (unifactoral model), the results of the multivariate analysis which included all the investigated SNPs indicated that rs2231375 is a risk factor for ccRCC (OR 1.47, 95% CI 1.08–2.01, *p* = 0.015) (Table 3).

### 2.3. Haplotype Analysis

The presence of a specific combination of SNPs may be essential for unveiling a distinctive phenotype; hence, haplotype analysis can aid in identifying such unique SNP compositions. Therefore, haplotype analysis was conducted on the whole group of patients seeking associations with ccRCC risk. We performed the haplotype analysis on the *HVEM* and *CD160* together, where haplotypes with frequencies below 3% were not considered. The results of the analysis are presented in Table 4. The haplotype analysis showed eight haplotypes with a frequency above 0.03. The global distributions of the haplotypes differed significantly between patients and HCs (χ^2^ = 20.33; df = 7; *p* = 0.005). Moreover, we noticed that the haplotype CAACT (rs1886730, rs2234167, rs8725, rs744877, rs2231375) was significantly overrepresented in patients (9.4% vs. 4.0%) and increased ccRCC risk (OR = 2.40; 95% CI 1.55–3.73; *p* = 5.78 × 10^−5^).

### 2.4. Sex-Dependent Association of HVEM and CD160 Polymorphisms and ccRCC Risk

As shown in Table 5, we observed a significant association between all except one (rs744877) of the SNPs investigated and ccRCC risk in females. Heterozygosity and/or possession of mutant alleles for rs1886730, rs2234167, rs8725, and rs2231375 increased the risk of disease compared to wild-type homozygotes. The increase in ccRCC odds was about 2.5-fold in the case of rs2234167 and rs8725 (*p* = 0.001; *p* = 0.01, respectively) and about 2-fold in the case of rs1886730 and rs2231375 (*p* = 0.02; *p* = 0.05, respectively).

### 2.5. Association of HVEM and CD160 Polymorphisms with Clinical Features of ccRCC

Based on available clinicopathological features, we subsequently conducted a stratified analysis relying on (1) cancer clinical stage, (2) presence of metastasis, (3) presence of necrosis, (4) patient age at diagnosis, and (5) size of the tumor.

We observed an association of heterozygosity with all SNPs except one (rs744877) with the clinical course of the disease (Tables S1–S5). In particular, we noticed that individuals possessing heterozygous genotypes are more prone to develop ccRCC, but this association is restricted to the early stages of disease (stage I or II) in all SNPs except one (for rs2234167, this association did not reach statistical significance). The same genotypes are related to higher odds of developing tumors smaller than 7 cm. However, these associations were not seen for bigger tumors and more advanced stages of disease. In addition, in patients younger than 63 years of age at diagnosis, individuals carrying the CT genotype for rs2231375 (*CD160*) were more prone to develop ccRCC.

### 2.6. Analysis of Patients’ Survival in Context of Clinical Parameters as Well as HVEM and CD160 Gene Polymorphisms

**Figure 1:**
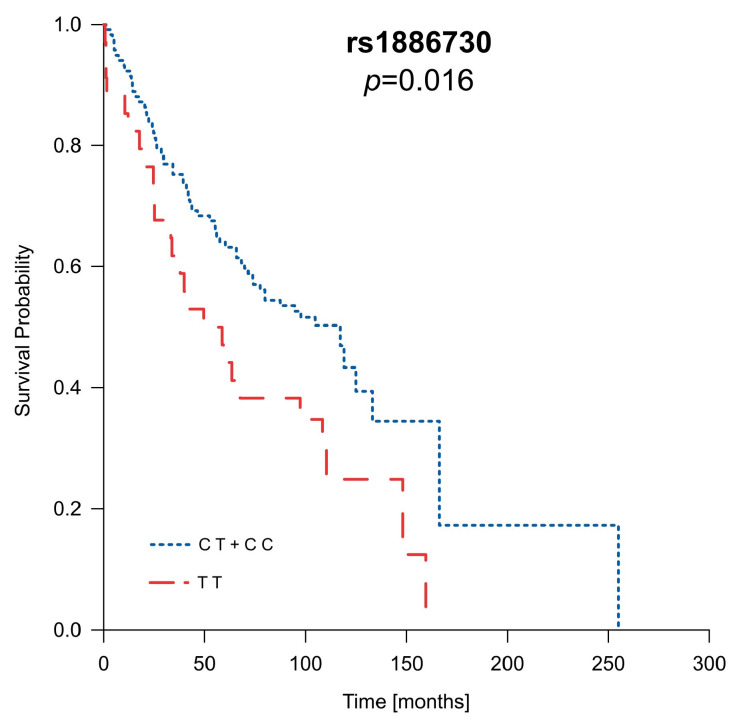
The rs1886730 variant of the *HVEM* gene predicts the overall survival in male ccRCC patients.

Polymorphisms in the *HVEM* and *CD160* genes, along with gender, age at diagnosis disease stage, tumor size, and the presence of metastasis or necrosis, were incorporated into the overall survival (OS) analysis. The outcomes of the OS analysis confirmed the impact of well-established risk factors like gender, disease stage, age at diagnosis, tumor size, and the presence of metastasis or necrosis on OS within our patient group (Table S6). In addition to these known risk factors, we observed that carriers of the C allele for rs1886730 (CT and CC genotypes) lived nearly twice as long as their counterparts (Table S7), and this difference approached statistical significance (*p* = 0.056). This influence of the rs1886730 variant was even more pronounced when considering the patient’s gender. Among males, the presence of the C allele in rs1886730 (CT and CC genotypes) significantly extended OS on average by 42 months (*p* = 0.016) (Figure 1 and Table S8). However, in female patients, rs1886730 did not impact their OS (Table S9). The results of the multivariate Cox proportional hazards model confirmed associations of the presence necrosis, metastasis, and rs1886730 TT genotype with poor prognosis for ccRCC patients.

## 3. Discussion

In recent years, there has been significant progress in identifying genetic risk factors for cancer, shedding light on specific gene mutations and variations associated with increased susceptibility to various cancers. Thanks to the result of the Human Genome Project, the understanding of genetic factors underlying familial cancers (which account for 10–30% of cancers) increased significantly [20]. About 100 genes (corresponding to 0.5% of all genes in the human genome) have highly or moderately penetrant mutations that underlie hereditary cancer syndromes. These include the major DNA mismatch repair (MMR) genes MLH1 and MSH2 causing Lynch syndrome (LS), the adenomatous polyposis coli (APC) gene prompting familial adenomatous polyposis (FAP), and the BRCA1 and BRCA2 genes that are strongly associated with hereditary breast and ovarian cancer syndromes [21]. However, in patients with sporadic disease, multiple common alleles may increase cancer risk, each individually having only a weak effect [22]. Understanding the genetic basis of cancer susceptibility is crucial for identifying individuals at higher risk, implementing preventive measures, and developing targeted interventions. For ccRCC, both genetic and epigenetic abnormalities lead to disease development and the generation of an altered proteome profile [23,24]. Molecular studies have shown that ccRCC is universally initiated by the biallelic inactivation of the Von Hippel–Lindau (VHL) gene [25], either due to somatic mutations or promoter hypermethylation [26]. Apart from VHL gene inactivation, other epigenetic genes also play a contributing role in ccRCC development, which mainly includes phosphatase and tensin homolog (PTEN) and breast cancer type-1 (BRCA-1)-associated protein-1 (BAP1) [23]. The latest study by Szegedi et al. confirmed that VHL or PTEN mutations may contribute to the development of human renal cancers [27].

Although mutations in a proto-oncogene, suppressor genes, and DNA repair genes are crucial for carcinogenesis, somatic mutations [28], as well as inherited variations in genes encoding molecules responsible for immunosurveillance, especially immune checkpoints, might be associated with cancer risk [8,29,30,31]. The IC receptor/ligand interaction in the tumor microenvironment is directly responsible for the impaired immune response against cancer and tumor cells’ evasion of immune surveillance. To prevent this process, in recent years, highly efficacious IC inhibitors (anti-CTLA-4 and anti-PD-1, alone or in combination), whose role is to enhance the immune response against cancer cells, were introduced into clinical practice, and they revolutionized cancer immunotherapy [32].

Inherited genetic variants present in the regulatory regions of the IC genes may be one of many factors responsible for the observed inter-individual differences in expression levels of ICs on immune cells. In addition, SNPs located in exons can introduce changes in the amino acid sequences of ICs, potentially affecting the functional properties of these molecules [16].

Since genetic variants can affect both the expression and structure of ICs, they are considered factors associated with cancer risk as well as cancer progression [33]. We showed in several previous studies [30,34,35,36] that variations in genes encoding the IC molecules are associated with the risk of developing ccRCC. In this present work’s context, it is interesting that polymorphisms in the gene encoding BTLA, another receptor that binds to the HVEM molecule and attenuates immune response, are associated with disease risk and progression. In particular, we showed that the rs1982809 SNP located within the 3’ intragenic region is associated with ccRCC risk [31]. Moreover, our study shows that the polymorphism rs1844089, located in the promoter region, influences the overall survival of ccRCC patients [34].

In this present study, we focused on the BTLA ligand HVEM and its other counterpart, CD160. The HVEM acts as a bidirectional molecular switch between activating (LIGHT and lymphotoxin LT-α) and inhibitory (BTLA) pathways, depending on the interacting receptor. Upon binding, the HVEM provides pro-survival and proliferative signals by activating nuclear transcription factor κB (NF-κB) and AKT transcriptional pathways, whereas BTLA attenuates T cell-mediated responses [8,9]. CD160 competes with BTLA for the same binding site within the complementarity-determining region (CDR) 1 of the HVEM, while LIGHT binds to the opposite side of the HVEM within CRD2/CRD3 regions. Therefore, inhibitory and stimulatory ligands of the HVEM bind at distinct sites [37,38]. Like the HVEM, CD160 is a dual-functioning signaling molecule, and the CD160/HVEM interaction induces different functions in different cell types. In T lymphocytes, the HVEM and CD160 interacting in a *trans* manner result in a coinhibitory signal that suppresses CD4+ T cell proliferation and IFN-γ production [39]. In contrast, in NK cells, the CD160/HVEM engagement delivers costimulatory signals that boost cytokine production and promote lytic activity, possibly via phosphorylation of AKT and ERK1/2 [40,41]. CD160 and HVEM are important regulators that exhibit multiple functional outcomes, which are sensitive to many variables, including competing ligands, expression patterns on different cell types, *cis* versus *trans* interactions, and the context-dependent direction of the signaling [42].

In this present work, we investigated the potential association between selected SNPs within *HVEM* and *CD160* genes and ccRCC risk and the clinical course of the disease. Analyzing the results for the HWE, we noticed that for rs2231375 within the *CD160* gene, the distribution of genotypes in the patient population is not in accordance with the HWE, but the frequency of genotypes in the control group is in the complete HWE. It is worth noting that genotype distribution in patients with disease would not be in the HWE as patient groups would not meet HW assumptions of a population—they are not representative of a general population, and selective pressure may act against variant alleles associated with diseases that reduce fitness. Moreover, the lack of the HWE may indicate an association between rs2231375 and ccRCC risk since, according to Lee et al. [43], deviation from the HWE of data sets of the affected individuals is sufficient to discover the relationship with disease. In fact, we observed statistically significant differences in the genotype distribution for rs2231375 between ccRCC patients and control individuals with overrepresentation of the heterozygotes in ccRCC. For all other SNPs, no deviation from the HWE was observed in either patients or controls. When analyzing the distribution of genotypes for the other SNPs investigated here, we noticed that possessing a minor allele for rs2234167 was associated with increased disease risk. Applying Svejgaard and Ryder analysis [19], we noticed that the heterozygosity of rs2234167 (*HVEM*) and rs2231375 (*CD160*) significantly increased the risk of ccRCC. That analysis also confirmed the independent association of both SNPs with ccRCC risk. However, multifactorial analysis indicated a stronger association for rs2231375 (*CD160*).

Interestingly, when considering the associations of *HVEM* and *CD160* SNPs with ccRCC risk in relation to gender, we noticed that the presence of a minor allele not only in the previously mentioned SNPs (rs2234167 (*HVEM*) and rs2231375 (*CD160*)) but also in two others (rs1886730 and rs8725, located within *HVEM*) was significantly associated with ccRCC risk in women. Neoplasms tend to be more prevalent in males, as evidenced by data from GLOBOCAN. This pattern is also observed in RCC, where there are 1.5 times as many cases in males compared to in females. This discrepancy may be attributed to the higher occurrence of lifestyle habits that promote carcinogenesis among men. The comprehensive VITamin and Lifestyle (VITAL) study conducted in the U.S. revealed that lifestyle factors such as obesity, hypertension, and smoking elevate the likelihood of RCC development. Furthermore, European findings indicate that high obesity, hypertension, and hyperglycemia levels in men, as well as high body mass index (BMI) in women, contribute to an increased risk of RCC [44]. Unfortunately, we lack lifestyle data for our patient and control groups, so it was impossible to adjust our results to the effects of smoking status, hypertension, and obesity, which could at least partially explain the different associations observed in men versus women.

Also of interest was the association between a higher risk of ccRCC in heterozygotes (rs1886730, rs2234167, rs8725, rs2231375) or the presence of a minor allele and clinical features of disease; however, it was observed only in the early stages of the disease, a smaller tumor size, and in individuals below 63 years of age at diagnosis. Such observations could indicate the impact of an aberrant HVEM/CD160 interaction (caused by gene variations) at the early stages of disease development. In contrast, at a later stage, the role of this pathway in regulating the immune response loses significance. However, since the subgroup analysis was performed on relatively small groups, the results should be treated with caution and confirmed on a larger cohort.

Haplotype analysis also indicated that the presence of a minor allele increased the risk of ccRCC since the CAACT haplotype (rs1886730, rs2234167, rs8725, rs744877, rs2231375) consisting of minor alleles increased the odds of ccRCC development more than 2-fold.

Very little is known about the functional relevance of the polymorphisms investigated here and their associations with cancer susceptibility. The presence of the rs2234167 SNP in the exon of the *HVEM* gene leads to amino acid substitution from isoleucine (A) to valine (G), but the biological consequence of that change is not described. So far, the rs2234167 polymorphism has been investigated in relation to esophageal squamous cell carcinoma (ESCC) risk in a relatively large group of Chinese patients (721 ESCC cases and 1208 controls). However, the authors found no association when analyzing the entire patient group, nor in the subgroup analyses based on stage, sex, age, BMI, smoking status, and alcohol consumption [18].

In the earlier work by Li et al. [17], the association between six *HVEM* SNPs (rs2281852, rs1886730, rs2234163, rs11573979, rs2234165, and rs2234167) and sporadic breast cancer was investigated. Contrary to our results, the authors observed a lower frequency of heterozygotes of rs2234167 in the breast cancer cases compared to in the control group. Also, opposite results were found for the rs1886730 SNP [17]. It is difficult to explain such different results, especially for women. However, as numerous meta-analyses indicate, the association of SNPs with cancer susceptibility varies in relation to ethnicity and cancer origin [45,46].

In our study, we also found an association of rs1886730 with the overall survival of ccRCC patients. What is interesting is that the presence of a minor allele significantly increased the susceptibility to ccRCC in women, while in men, it is associated with prolonged survival. Introns have been found to play various functional roles. By altering splice site recognition or by modulating the binding of splicing regulatory proteins, introns impact the binding affinity of transcription factors or other regulatory proteins and microRNA binding sites, hence affecting genomic stability and leading to structural variations such as insertions, deletions, or chromosomal rearrangements. Consequently, variations within introns may affect gene expression, splicing patterns, or other regulatory processes implicated in disease pathogenesis [47]. Since little is known about that intronic variant of the *HVEM* gene, it is hard to speculate about its function in terms of explaining the phenomena observed here.

*HVEM/LIGHT/BTLA/CD160* polymorphisms were also investigated in the context of the occurrence of antibody-mediated rejection (ABMR) in renal transplant recipients [48]. The authors investigated 17 SNPs within the *HVEM* gene (rs2234167 and rs8725 among others), 17 SNPs within the *LIGHT* gene, and 6 SNPs within the *CD160* gene (including rs2231375). The results of that study showed that none of the studied 41 *HVEM/LIGHT/BTLA/CD160* gene polymorphisms were associated with ABMR.

As we stated earlier, very limited data regarding CD160 SNPs are available. Except for the above-mentioned publication related to rejection in renal transplant, only one work was devoted to the association between *CD160* rs744877 and rs3766526 SNPs and autoimmune thyroid disease (AITD) [49]. The authors noticed a significant association between rs744877 and AITD. However, in our study, we did not find any association of that polymorphism in the risk analysis in the whole group or subgroups, nor any association with patients’ overall survival.

A limitation of our study is that our patients were recruited from a single center, and therefore, our treatment results are representative of a tertiary cancer center. Consequently, there is an overrepresentation of stage IV patients in our population, which is characteristic of tertiary cancer centers. Moreover, data about other RCC risk factors like smoking status and alcohol consumption, as well as the same data for controls, were unavailable and were not included in multivariable analysis. Another limitation is the mismatched age of patients and controls. We realize that healthy individuals could develop cancer in the future. However, the incidence of renal cell cancer is about 1 per 10,000 cases (different in men and women). Given this, the chance that there would be a number of people in the control group at a later age that would distort the obtained results is small. Another limitation was the inability to discuss our results regarding their functional role on mRNA stability, expression, epigenetic control, and protein expression. However, the current knowledge on that subject is very limited, and we do not have arguments for such a discussion. Additional research into the underlying mechanisms influenced by specific SNPs must be elicited to confirm our findings. Finally, a study on a larger group of patients and other populations is needed since our subgroup analyses were carried out on a relatively small patient cohort.

## 4. Materials and Methods

### 4.1. Patients

This study comprised 238 ccRCC consecutively recruited patients diagnosed between 2009 and 2012 at the Department of Urology and Oncologic Urology at Wroclaw Medical University. Samples were collected from 2010 to 2012. The research involving human participants underwent thorough review and approval by the Bioethical Committee of Wroclaw Medical University. The DNA utilized in this study was isolated from patients initially recruited for a prior project approved by the Ethics Committee of Wroclaw Medical University (KB 55/2010). Subsequently, for the current study and the reuse of materials, additional approvals were obtained from the Ethics Committee (KB 587/2020 and KB 755/2022). This study was conducted in accordance with the Helsinki Declaration. All participants provided their written informed consent to participate in this study. Overall survival was evaluated from the date of diagnosis to the date of death from any cause or up to 24 January 2020, the completion date for data collection. Patient characteristic is presented in Table 6. 

### 4.2. Controls

The control group included 521 individuals (349 males and 172 females) selected from the identical geographic region as ccRCC patients. The median age of controls was 29; Q1–Q3: 23–38 range 18–63 (for women—median 29; Q1–Q3: 23–38 range 18–61; for men—28; Q1–Q3: 23–38 range 18–63). Blood samples from the healthy subjects were obtained either from the Wroclaw Blood Bank or through donations from employees of the Ludwik Hirszfeld Institute of Immunology and Experimental Therapy. The research involving human participants underwent thorough review and received approval from the Bioethical Committee of Wroclaw Medical University in Wroclaw, Poland (as mentioned above). The study was conducted in accordance with the Helsinki Declaration. All participants in this study provided written informed consent to participate.

### 4.3. Selection of SNPs

Among the five selected single nucleotide polymorphisms (SNPs), two, namely rs1886730 (*HVEM*) and rs2234167 (*HVEM*), were investigated in a previous study focusing on cancer susceptibility. Another SNP, rs744877 (*CD160*), was examined in the context of autoimmune thyroid disease and the risk of rheumatoid arthritis. Additionally, for this study, we identified and included two new SNPs that had not been previously studied: rs8725 (*HVEM*) (located in 3’ UTR) and rs2231375 (*CD160*) (intron variant/3’UTR variant), utilizing the UCSC database (https://genome.ucsc.edu/index.html; accessed on 9 January 2023). Comprehensive details regarding the localization and additional information for each SNP are outlined in provided Table S10.

### 4.4. DNA Isolation and SNP Genotyping

Genomic DNA was isolated from refrozen blood samples by GeneMATRIX Quick Blood DNA Purification Kit (EURX, Gdansk, Poland) according to the manufacturer’s instructions. SNPs were genotyped using Probe qPCR Master Mix (2x) plus ROX solution (EURX, Gdansk, Poland) and TaqMan assays (Catalog # 4351379; Applied Biosystems. Foster City, USA); rs1886730 (*HVEM*) assay ID: C__11448257_10; rs2234167 (*HVEM*) assay ID: C__16181162_10; rs8725 (*HVEM*) assay ID: C__8861318_10; rs744877 (*CD160*) assay ID: C__1862234_10; and rs2231375 (*CD160*) assay ID: C__15854941_10. All reactions were run on the ViiA7 Real-Time PCR system (Applied Biosystems, Singapore, Singapore). Data were analyzed by the software Quant Studio ^TM^ Real Time PCR Software v1.3.

### 4.5. Statistical Analysis

Statistical analysis was conducted utilizing SigmaPlot 11.0 software (Systat Software, San Jose, CA, USA). For measurable variables, means, medians, and standard deviations were calculated. The Hardy–Weinberg equilibrium (HWE) for all genotyped *HVEM* and *CD160* SNPs was evaluated independently for both ccRCC patients and healthy controls. This evaluation was carried out by comparing observed and expected genotype frequencies using the χ2 test. The χ2 test was also used to compare categorical data between ccRCC patients and controls. To assess the relationship between the studied polymorphisms and susceptibility to ccRCC, odds ratios (ORs) and 95% confidence intervals (95% CIs) were calculated using the binary logistic regression model. To assess the significance of the potential influence of two observed genetic factors associated with ccRCC risk, the Svejgaard and Ryder test was implemented [19].

Haplotype frequencies for pairs of alleles were determined using the online software SHEsis (http://analysis.bio-x.cn/myAnalysis.php; accessed on 4 December 2023), excluding haplotypes with frequencies below 0.01. Survival analysis (OS) was conducted utilizing the Kaplan–Meier estimator with SigmaPlot 11.0 software (Systat Software, San Jose, CA, USA). Patient survival was compared against selected clinical variables using the log-rank test. Statistical significance was considered for differences between groups if *p* < 0.05.

The impact of selected factors on the occurrence of change was analyzed using logistic regression (a unifactorial model of predictors included in the analysis). Odds ratios and confidence intervals were determined. The next step involved constructing a multifactorial model, considering the variables where the *p*-value did not exceed 0.30. The model was evaluated using a set of standard goodness-of-fit measures (AIC, BIC, Hosmer–Lemeshow test).

To assess the impact of qualitative or quantitative variables on survival, the Cox proportional hazards model was used. The model-building process was conducted using a stepwise method, and the model evaluation employed a set of standard goodness-of-fit measures (AIC, R2). Hazard ratios (HRs) were considered statistically significant at *p* < 0.05.

## 5. Conclusions

In conclusion, our study showed an association of *HVEM* and *CD160* polymorphisms with ccRCC risk and overall survival. This association was noticed especially when considering the receptor/ligand SNP combination of rs2234167 within the *HVEM* and rs2231375 in *CD160*. Moreover, rs1886730, rs2234167, rs8725, and rs2231375 were associated with ccRCC risk in women. Additionally, rs1886730 variations were associated with ccRCC patients’ overall survival, especially in male patients. Nevertheless, studies with larger patient groups and functional evaluation of studied *HVEM* and *CD160* polymorphisms are needed to confirm our findings.

## Figures and Tables

**Table 1:** Genotype frequencies of *HVEM* and *CD160* polymorphisms and their associations with ccRCC risk.

SNP	Genotype	Case		Control		OR (95% CI)	*p* Value *
		N	%	N	%		
**rs1886730T > C**	TT	49	20.59	140	26.87	1	0.17
	CT	130	54.62	257	49.33	1.44 (0.98–2.12)	
	CC	59	24.79	124	23.80	1.36 (0.87–2.12)	
	CT + CC	189	79.41	381	73.13	1.41 (0.98–2.04)	0.06
	TT + CT	179	75.21	397	76.20	0.94 (0.66–1.35)	0.77
**rs2234167G > A**	GG	167	70.17	404	77.54	1	**0.06**
	AG	68	28.57	108	20.73	1.52 (1.07–2.17)	
	AA	3	1.26	9	1.73	0.89 (0.26–3.07)	
	AG + AA	71	29.83	117	22.46	1.47 (1.04–2.07)	**0.03**
	GG + AG	235	98.74	512	98.27	1.25 (0.36–4.29)	0.63
**rs8725G > A**	GG	52	21.85	145	27.83	1	0.22
	AG	125	52.52	252	48.37	1.38 (0.94–2.02)	
	AA	61	25.63	124	23.80	1.37 (0.88–2.12)	
	AG + AA	186	78.15	376	72.17	1.37 (0.96–1.97)	0.08
	GG + AG	177	74.37	397	76.20	0.90 (0.64–1.29)	0.59
**rs744877C > A**	CC	77	32.35	173	33.21	1	0.95
	AC	118	49.58	258	49.52	1.03 (0.73–1.45)	
	AA	43	18.07	90	17.27	1.08 (0.69–1.69)	
	AC + AA	161	67.65	348	66.79	1.04 (0.75–1.44)	0.82
	CC + AC	195	81.93	431	82.73	0.94 (0.63–1.40)	0.79
**rs2231375C > T**	CC	78	33.05	206	39.62	1	**0.03**
	CT	130	55.08	233	44.81	1.47 (1.05–2.06)	
	TT	28	11.86	81	15.58	0.92 (0.56–1.52)	
	CT + TT	158	66.95	314	60.38	1.33 (0.96–1.83)	0.08
	CC + CT	208	88.14	439	84.42	1.36 (0.86–2.14)	0.18

* significant associations were bolded.

**Table 2:** Analysis of the associations between two genetic factor carriers of the *HVEM* rs2243167 [AG] genotype and the *CD160* 2,231,375 [CT] genotype with ccRCC risk using the Svejgaard and Ryder method.

	ccRCC (n = 238)		Control (n = 521)		
A+, B+	39		57		
A+, B−	28		51		
A−, B+	91		177		
A−, B−	78		236		
**Test**	**OR**	*p* value *	**95% CI**	**Comparison**	**Individual association**
(a) A+	1.53	0.02	1.08–2.18		
(b) B+	1.58	0.04	1.16–2.15		
(c) A+ B+ vs. A−B+	1.33	0.24	0.82–2.15	A in B positive	A association
(d) A+ B− vs. A −B −	1.85	**0.02**	1.09–3.13	A in B negative	
(e) A+ B+ vs. A+ B−	1.25	0.48	0.67–2.31	B in A positive	B association
(f) A− B + vs. A − B−	1.56	**0.02**	1.09–2.23	B in A negative	
(g) A + B− vs. A− B+	1.07	0.81	0.63–1.81	Differences between A and B	
(h) A +B + vs. A− B−	2.07	**0.003**	1.30–3.35	Combined association	

* significant associations were bolded.

**Table 3:** Unifactorial and multifactorial logistic regression analysis of an association of *HVEM* and CD160 gene variations with ccRCC risk.

Logistic Regression		Regression Coefficient	Standard Error	*p*-Value *	OR	CI 95%
**Unifactorial Model**						
rs2234167	AA + AG	0.38	0.18	**0.029**	1.47	1.04–2.07
rs8725	G G	−0.32	0.18	0.082	0.72	0.50–1.04
rs1886730	T T	−0.35	0.19	0.064	0.71	0.49–1.02
rs2231375	C T	0.41	0.16	**0.010**	1.50	1.10–2.05
**Multifactorial Model**						
rs2234167	AA +AG	0.26	0.19	0.16	1.30	0.90–1.88
rs8725	G G	−0.09	0.33	0.80	0.92	0.48–1.75
rs1886730	T T	−0.18	0.34	0.60	0.84	0.43–1.62
rs2231375	C T	0.39	0.16	**0.015**	1.47	1.08–2.01

* significant associations were bolded.

**Table 4:** The haplotype distribution of studied SNPs between ccRCC patients and controls.

Haplotype *	Case (%)	Control (%)	Odds Ratio [95% CI]	*p* Value **
CAAAC	15.23 (0.032)	41.05 (0.039)	0.79 [0.434~1.438]	0.44
CAACT	44.30 (0.094)	42.11 (0.040)	2.41 [1.55~3.73]	**5.78 × 10^−5^**
CGAAC	79.55 (0.179)	156.46 (0.150)	1.11 [0.83~1.50]	0.48
CGACC	29.73 (0.063)	83.12 (0.080)	0.75 [0.49~1.16]	0.20
CGACT	50.36 (0.107)	111.32 (0.107)	0.97 [0.68~1.38]	0.86
TGGAC	92.68 (0.196)	189.24 (0.182)	1.07 [0.81~1.41]	0.65
TGGCC	39.02 (0.083)	102.89 (0.099)	0.80 [0.54~1.17]	0.25
TGGCT	80.71 (0.171)	201.04 (0.193)	0.83 [0.62~1.11]	0.21
Global χ2 = 20.33, df = 7, *p* = **0.005**				

* rs1886730, rs2234167, rs8725, rs744877, rs2231375; ** significant associations were bolded.

**Table 5:** The analysis between rs1886730, rs2234167, rs8725 (*HVEM*), and rs2231375 (*CD160*) and ccRCC risk in females.

	N (Case/Control)			OR (95% CI); *p* Value *							
**rs1886730**	TT	CT	CC	CT + CC vs. TT		CT + TT vs. CC		CT vs. TT		CT vs. TT + CC	
	15/50	46/77	25/44	1.92 (1.01–3.64);	**0.04**	0.84 (0.47–1.49);	0.57	1.95 (0.99–3.84);	**0.05**	1.40 (0.83–2.36);	0.20
**rs2234167**	GG	AG	AA	AG + AA vs. GG		GG + AG vs. AA		AG vs. GG		AG vs. GG + AA	
	55/142	37/80	1/2	2.63 (1.46–4.73);	**0.001**	0.84 (0.11–6.47);	1.00	2.73 (1.50–4.96);	**0.001**	2.76 (1.51–5.02);	**0.001**
**rs8725**	GG	AG	AA	AG + AA vs. GG		GG + AG vs. AA		AG vs. GG		AG vs. GG + AA	
	14/56	47/74	25/41	2.45 (1.28–4.67);	**0.01**	0.77 (0.43–1.37);	0.38	2.48 (1.26–4.91);	**0.01**	1.58 (0.94–2.66);	0.09
**rs2231375**	CC	CT	TT	TT + CT vs. CC		CC + CT vs. TT		CT vs. CC		CT vs. CC + TT	
	28/77	47/65	10/28	1.67 (0.97–2.87);	0.06	1.44 (0.67–3.08);	0.32	1.97 (1.12–3.48);	**0.02**	2.00 (1.18–3.39);	**0.01**

* significant associations were bolded.

**Table 6:** Patient characteristics.

Variable	All N = 238	Male N = 151	Female N = 86
**Age at diagnosis**			
Median	62	61	63
Mean	62.61	62.01	63.67
Q1–Q3	56–70	56–68	58–71
Min, Max	21, 85	21, 85	24, 85
**BMI**			
Median	27.70	27.70	27.75
Mean	28.29	28.26	28.33
Q1–Q3	24.6–31.5	25.1–30.7	23.85–31.2
Min, Max	19.1, 43.8	19.7, 43.8	19.1, 43.8
**Stage of disease**	**N** (%)	**N** (%)	**N** (%)
I	108 (45.57)	63 (41.72)	45 (52.33)
II	26 (10.97)	20 (13.25)	6 (6.98)
III	26 (10.97)	16 (10.60)	10 (11.63)
IV	76 (32.07)	51 (33.77)	25 (29.07)
Unknown	1 (0.42)	1 (0.66)	0 (0)
**Metastasis**			
No	165 (69.62)	101 (66.89)	64 (74.42)
Present	53 (22.36)	35 (23.18)	18 (20.93)
Unknown	19 (8.02)	15 (9.93)	4 (4.65)
**Necrosis**			
No	118 (59.00)	71 (55.47)	47 (65.28)
Present	82 (41.00)	57 (44.53)	25 (34.72)
Unknown	0 (0)	0 (0)	0 (0)
**Tumor size**			
<70 mm	143 (60.34)	87 (57.61)	56 (65.12)
>70 mm	65 (27.42)	48 (31.79)	17 (19.77)
Unknown	29 (12.24)	16 (10.60)	13 (15.11)

## Data Availability

The original results are presented in the article and Supplementary Materials. Additional data are available upon reasonable request from the corresponding author.

## Supplementary Materials

The following supporting information can be downloaded at https://www.mdpi.com/article/10.3390/ijms25136860/s1.
