# Supplementary material for: Effect of HVEM/CD160 Variations on the Clear Cell Renal Carcinoma Risk and Overall Survival
Source: Int J Mol Sci. 2024 Jun 22;25(13):6860. doi: 10.3390/ijms25136860 (PMC11241222; doi:10.3390/ijms25136860)
Supplement: Supplementary file 1 [file ijms-25-06860-s001.zip › ijms-3055653-supplementary.pdf]

SUPPLEMENT

Table S1 Stratified analysis between rs1886730 (*HVEM*) and ccRCC risk by stage, age, and tumor size

Variable	rs1886730 C > T			OR (95% CI); *p value*				
	**TT**	**CT**	**CC**	**CT+CC vs TT**	**CT+TT vs CC**	**CT vs TT**	**CT vs CC**	**Allele C vs Allele T**
		N (case)						
**Control**	140	180	80	1	1	1	1	1
**Stage**								
I and II	25	81	43	1.80 (1.13-2.87); **0.01**	0.77 (0.51-1.15); 0.21	1.74 (1.07-2.85); **0.02**	0.77 (0.51-1.15); 0.66	1.35 (1.05-1.75); **0.02**
III and IV	22	45	15	0.99 (0.59-1.67); 0.99	1.36 (0.76-2.45); 0.27	1.10 (0.64-1.90); 0.70	1.36 (0.76-2.45); 0.24	0.90 (0.65-1.25); 0.52
**Age**								
< 63	30	76	26	1.24 (0.79-1.94); 0.33	1.26 (0.79-2.02); 0.32	1.37 (0.86-2.18); 0.18	1.26 (0.79-2.02); 0.17	1.00 (0.76-1.31); 1.00
> 63	19	51	31	1.56 (0.92-2.65); 0.09	0.70 (0.44-1.12); 0.14	1.44 (0.82-2.52); 0.19	0.70 (0.44-1.12); 0.36	1.35 (1.00-1.82); 0.05
**Tumor size**								
< 70 mm	20	72	34	1.91 (1.15-3.19); **0.01**	0.84 (0.54-1.30); 0.46	1.93 (1.13-3.28); **0.01**	0.84 (0.54-1.30); 0.93	1.33 (1.01-1.75); **0.04**
> 70 mm	20	46	16	1.12 (0.66-1.92); 0.64	1.26 (0.71-2.24); 0.39	1.24 (0.71-2.16); 0.43	1.26 (0.71-2.24); 0.29	0.96 (0.69-1.34); 0.83

Table S2 Stratified analysis between rs2234167 (*HVEM*) and ccRCC risk by stage, age, and tumor size

Variable	rs2234167 G > A			OR (95% CI); *p value*				
	**GG**	**AG**	**AA**	**AA+AG vs GG**	**GG+AG vs AA**	**AG vs GG**	**AG vs AA**	**Allele A vs Allele G**
		N (case)						
**Control**	404	108	9	1	1	1	1	1
**Stage**								
I and II	105	42	3	1.45 (0.97-2.18); 0.08	0.78 (0.22-2.68); 0.82	1.47 (0.97-2.23); 0.08	0.78 (0.22-2.68); 0.85	1.37 (0.95-1.96); 0.09
III and IV	58	24	0	1.44 (0.86-2.41); 0.18	3.06 (0.18-3.05); 0.23	1.56 (0.93-2.62); 0.10	3.06 (0.18-53.05); 0.16	1.26 (0.79-2.02); 0.36
**Age**								
< 63	93	38	1	1.45 (0.95-2.22); 0.09	1.63 (0.29-9.19); 0.42	1.54 (0.99-2.36); 0.05	1.63 (0.29-9.19); 0.26	1.31 (0.89-1.92); 0.18
> 63	70	29	2	1.54 (0.96-2.46); 0.08	0.74 (0.18-3.02); 0.86	1.56 (0.97-2.52); 0.07	0.74 (0.18-3.02); 0.82	1.43 (0.95-2.17); 0.10
**Tumor size**								
< 70 mm	84	40	2	1.73 (1.14-2.64); **0.01**	0.92 (0.23-3.77); 0.91	1.79 (1.16-2.75); **0.01**	0.92 (0.23-3.77); 0.52	1.55 (1.06-2.25); **0.02**
> 70 mm	59	22	1	1.36 (0.81-2.29); 0.27	1.01 (0.18-5.73); 0.74	1.41 (0.83-2.39); 0.22	1.01 (0.18-5.73); 0.57	1.26 (0.79-2.02); 0.36

Table S3 Stratified analysis between rs8725 (*HVEM*) and ccRCC risk by stage, age, and tumor size

Variable	rs8725 G > A			OR (95% CI); *p value*				
	**GG**	**AG**	**AA**	**AA+AG vs GG**	**GG+AG vs AA**	**AG vs GG**	**AG vs AA**	**Allele A vs Allele G**
		N (case)						
**Control**	145	252	124	1	1	1	1	1
**Stage**								
I and II	26	79	44	1.80 (1.14-2.86); **0.01**	0.74 (0.50-1.11); 0.16	1.73 (1.06-2.81); **0.02**	0.74 (0.50-1.11): 0.57	1.38(1.07-1.79); **0.01**
III and IV	23	43	16	0.98 (0.58-1.64); 0.97	1.26 (0.71-2.24); 0.40	1.07 (0.62-1.83); 0.80	1.26 (0.71-2.24); 0.37	0.91 (0.66-1.27); 0.59
**Age**								
< 63	32	71	29	1.20 (0.77-1.85); 0.41	1.10 (0.70-1.73); 0.66	1.27 (0.80-2.01); 0.30	1.10 (0.70-1.73); 0.45	1.04 (0.79-1.36); 0.80
> 63	20	51	30	1.54 (0.91-2.59); 0.10	0.73 (0.46-1.17); 0.21	1.45 (0.83-2.51); 0.18	0.73 (0.46-1.17); 0.48	1.32 (0.98-1.79); 0.07
**Tumor size**								
< 70 mm	22	69	35	1.79 (1.10-2.94); **0.02**	0.81 (0.52-1.25); 0.35	1.78 (1.06-2.99); **0.03**	0.81 (0.52-1.25); 0.90	0.81 (0.52-1.25); 0.35
> 70 mm	21	44	17	1.11 (0.65-1.87); 0.68	1.17 (0.67-2.06); 0.54	1.19 (0.69-2.07); 0.51	1.17 (0.67-2.06); 0.43	0.98 (0.71-1.37); 0.92

Table S4 Stratified analysis between rs2231375 (*CD160*) and ccRCC risk by stage, age, and tumor size

Variable	rs2231375 C > T			OR (95% CI); *p value*				
	**CC**	**CT**	**TT**	**TT+CT vs CC**	**CC+CT vs TT**	**CT vs CC**	**CT vs TT**	**Allele T vs Allele C**
		N (case)						
**Control**	206	233	81	1	1	1	1	1
**Stage**								
I and II	48	81	20	1.37 (0.94-2.02); 0.10	1.17 (0.69-1.98); 0.52	1.49 (0.99-2.22); **0.05**	1.17 (0.69-1.98); 0.22	1.12 (0.86-1.45); 0.41
III and IV	26	47	7	1.35 (0.82-2.22); 0.22	1.82 (0.83-3.99); 0.11	1.59 (0.95-2.64); 0.07	1.82 (0.83-3.99); **0.04**	1.01 (0.72-1.42); 0.97
**Age**								
< 63	37	77	17	1.65 (1.09-2.51); **0.02**	1.21 (0.70-2.12); 0.46	1.83 (1.19-2.82); **0.01**	1.21 (0.70-2.12); 0.13	1.20 (0.91-1.58); 0.19
> 63	40	49	11	0.98 (0.63-1.52); 0.94	1.44 (0.75-2.79); 0.24	1.08 (0.69-1.70); 0.73	1.44 (0.75-2.79); 0.22	0.90 (0.66-1.23); 0.51
**Tumor size**								
< 70 mm	38	74	13	1.49 (0.98-2.27); 0.06	1.55 (0.84-2.85); 0.14	1.71 (1.11-2.64); **0.01**	1.55 (0.84-2.85); **0.04**	1.09 (0.82-1.44); 0.56
> 70 mm	30	39	12	1.11 (0.69-1.79); 0.66	1.03 (0.54-1.97); 0.86	1.15 (0.69-1.90); 0.59	1.03 (0.54-1.97); 0.73	1.04 (0.74-1.46); 0.83

Table S5 Stratified analysis between rs744877 (*CD160*) and ccRCC risk by stage, age, and tumor size

Variable	rs744877 C > A			OR (95% CI); *p value*				
	**CC**	**AC**	**AA**	**AA+AC vs CC**	**CC+AC vs AA**	**AC vs CC**	**AC vs AA**	**Allele C vs Allele A**
		N (case)						
**Control**	173	258	90	1	1	1	1	1
**Stage**								
I and II	51	71	27	0.95 (0.65-1.40); 0.82	0.93 (0.58-1.50); 0.81	0.93 (0.62-1.40); 0.74	0.93 (0.58-1.50); 0.74	1.00 (0.77-1.29); 0.98
III and IV	25	42	15	1.12 (0.68-1.85); 0.63	0.91 (0.50-1.66); 0.82	1.12 (0.66-1.90); 0.66	0.91 (0.50-1.66); 0.94	1.08 (0.78-1.50); 0.63
**Age**								
< 63	42	69	21	1.06 (0.71-1.59); 0.76	1.09 (0.65-1.82) 0.71	1.10 (0.72-1.68); 0.66	1.09 (0.65-1.82); 0.62	1.00 (0.76-1.32); 1.00
> 63	34	45	22	0.97 (0.62-1.53); 0.93	0.74 (0.44-1.25); 0.28	0.89 (0.55-1.43); 0.59	0.74 (0.44-1.25); 0.24	1.09 (0.80-1.47); 0.60
**Tumor size**								
< 70 mm	41	62	23	1.03 (0.68-1.55); 0.89	0.92 (0.56-1.53); 0.80	1.01 (0.65-1.56); 0.95	0.92 (0.56-1.53); 0.82	1.04 (0.78-1.37); 0.81
> 70 mm	29	36	17	0.90 (0.56-1.47); 0.70	0.79 (0.44-1.39); 0.45	0.83 (0.49-1.40); 0.49	0.79 (0.44-1.39); 0.34	1.03 (0.74-1.43); 0.88

Table S6 Probability of survival in relation to clinical features

Variable		Median	Mean	SD	Survived [n]	Death [n]	*p value*
**Gender**	Female	126.87	146.29	17.66	47	38	0.036
	Male	94.53	98.11	9.49	62	89	
**Age**	< 63	119.03	127.99	13.95	63	58	0.030
	> 63	74.07	109.16	13.22	45	67	
**Metastasis**	No	146.13	140.27	11.89	95	70	<0.001
	Present	26.37	42.90	6.16	8	45	
**Necrosis**	No	-	183.01	9.99	75	42	<0.001
	Present	57.50	95.70	13.42	25	57	
**Stage**	1	166.27	138.40	9.51	70	37	<0.001
	2	-	93.72	10.86	16	10	
	3	108.47	91.22	11.64	12	14	
	4	39.87	70.57	9.42	11	65	
**Tumor size**	< 70 mm	117.17	106.79	5.67	80	63	<0.001
	> 70 mm	41.00	62.09	6.29	18	47	

Table S7 Probability of survival in relation to the *HVEM* and *CD160* gene polymorphisms in the whole group of patients

Variable		Median	Mean	SD	Survived [n]	Death [n]	*p value*
**rs1886730**	TT	67.63	96.33	15.25	33	16	0.056
	CT+CC	119.03	122.61	11.64	94	93	
**rs2234167**	GG	104.77	112.25	9.84	94	72	0.440
	AG+AA	133.23	137.05	22.75	33	37	
**rs8725**	GG	82.30	97.97	14.93	34	18	0.109
	AG+AA	117.17	123.58	11.87	93	91	
**rs744877**	CC	104.77	89.10	6.56	41	36	0.607
	AC+AA	110.40	121.92	10.78	86	73	
**rs2231375**	CC	117.17	114.30	13.90	41	37	0.795
	CT+TT	104.77	119.35	12.28	84	72	

Table S8 Probability of survival in relation to the *HVEM* and *CD160* gene polymorphisms in male patients

**Variable**		**Median**	**Mean**	**SD**	**Survived [n]**	**Death [n]**	***p value***
**rs1886730**	TT	49.63	70.82	10.11	26	8	**0.016**
	CT+CC	117.17	112.81	14.15	63	54	
**rs2234167**	GG	87.53	98.96	10.26	68	44	0.926
	AG+AA	97.63	81.51	8.76	21	18	
**rs8725**	GG	59.33	76.06	9.68	27	11	0.086
	AG+AA	97.63	112.83	14.50	62	51	
**rs744877**	CC	104.77	85.94	7.34	26	22	0.710
	AC+AA	79.87	97.13	10.62	63	40	
**rs2231375**	CC	67.63	88.84	9.51	30	20	0.733
	CT+TT	97.63	102.83	12.75	58	42	

Table S9 Probability of survival in relation to the *HVEM* and *CD160* gene polymorphisms in female patients

**Variable**		**Median**	**Mean**	**SD**	**Survived [n]**	**Death [n]**	***p value***
**rs1886730**	TT	-	158.36	31.75	7	8	0.985
	CT+CC	126.87	135.89	21.67	31	39	
**rs2234167**	GG	126.87	135.67	19.80	26	28	0.454
	AG+AA	264.73	183.01	20.64	12	19	
**rs8725**	GG	93.07	151.15	32.93	7	7	0.810
	AG+AA	126.87	136.86	21.83	31	40	
**rs744877**	CC	146.13	91.53	11.41	15	14	0.158
	AC+AA	126.87	163.91	20.24	23	33	
**rs2231375**	CC	126.87	143.98	24.87	11	17	0.395
	CT+TT	264.73	163.57	15.44	26	30	

Table S10 Characteristics of *HVEM* and *CD160* polymorphisms

**Position** (GRCh38.p14)	**Gene**	**SNP**	**Variation**	**Gene region**	**TaqMan Assay**
chr1:2557169	*HVEM*	rs1886730	T>C	Intron Variant	C__11448257_10
chr1:2562891	*HVEM*	rs2234167	G>A, V(Val)>I(Ile)	Missense Variant	C__16181162_10
chr1:2563346	*HVEM*	rs8725	G>A	3’UTR Variant	C___8861318_10
chr1:145720709	*CD160*	rs744877	C>A	Intron Variant	C___1862234_10
chr1:145738392	*CD160*	rs2231375	C>T	Intron Variant	C__15854941_10
